# Sustainable Valorization of *Litsea cubeba* (Lour.) Pers. Residue as the New Lauric Oil Source Using Alternative Green Extraction and Refining Methods

**DOI:** 10.3390/foods11142047

**Published:** 2022-07-11

**Authors:** Ying Li, Xiaoci Zhuang, Xinrui Wu, Chaoying Qiu, Yong Wang

**Affiliations:** 1 Guangdong International Joint Research Center for Oilseeds Biorefinery, Nutrition and Safety, Department of Food Science and Engineering, Jinan University, Guangzhou 510632, China; yingli@jnu.edu.cn (Y.L.); zhuangxiaoci@stu2016.jnu.edu.cn (X.Z.); tcqiu@jnu.edu.cn (C.Q.); 2 International College, Jinan University, Guangzhou 510632, China; surire29@163.com; 3 Qingyuan Yaokang Biotechnology, Qingyuan 513200, China

**Keywords:** *Litsea cubeba*, sustainable valorization, lipid extraction, green extraction, lauric oils, oil refining

## Abstract

*Litsea cubeba* is an ethnic woody oil plant, in which essential oil rather than oil has been the main foreign trade product through the decades. Concerning large amounts of residue generated from *L. cubeba* essential oil processing, a sustainable valorization pathway of these biowastes is proposed in this study. First, such biowastes have been systematically investigated for the first time regarding their oils extracted by three extraction methods, where ultrasound-assisted extraction (UAE) could significantly improve the extraction rate of traditional pressing and solvent extraction without any changes in oil quality. Moreover, the composition of acylglycerols and fatty acids in *L. cubeba* fruit, kernel, and peel oils were also first identified, which further proved that peels with abundant free fatty acids could lead to high acid value of *L. cubeba* fruit oils. Compared to virgin coconut oils, *L. cubeba* kernel oils have a more balanced fatty acid composition with a high lauric acid level, which could be applied as a promising lauric oil resource. Considering the high acid value in *L. cubeba* kernel oils, both decoloration using activated clay and alkali deacidification were attempted, where the combination of alkali deacidification and 10% of activated clay performed the best considering both quality and cost.

## 1. Introduction

*Litsea cubeba* (Lour.) Pers. belonging to the Lauraceae family is a unique economic woody oil plant in China and Southeast Asia. Its essential oil mainly containing citral, limonene and some other aromatic hydrocarbons are renowned worldwide, which can be extracted from its flower, leaf, fruit, stem, and root by hydrodistillation [1]. China possesses abundant *L. cubeba* resources, which is also the world’s largest producer and exporter for *L. cubeba* essential oils over decades. Such lemony essential oil could not only be used as a natural synthon for high valued-added citral-derived fragrances and vitamin A, but also be widely applied as formula ingredients in food, cosmetic, personal, and pet care products due to its broad-spectrum bioactivities [2,3,4,5]. Since the yield of *L. cubeba* essential oils is limited, a large number of residues generated after processing has gradually become an intractable environmental burden that must be solved [6]. Actually, apart from kernel residues after the *L. cubeba* essential oil production, there are also enormous dried fruit residues generated due to limited capacity of both production and storage, which are usually burned as firewoods or discarded directly to the environment. Assuming the average yield of *L. cubeba* essential oil is 5%, the annual output of *L. cubeba* in China is around 30,000–40,000 tons according to incomplete statistics, leading to around 28,500–38,000 tons of residues generated. Although several recent studies tried to use them for either animal feed or oil pressing and modification for the sake of improving utilization rate [7,8,9], the integrated utilization of *L. cubeba* toward a sustainable valorization still need progress in exploring appropriate techniques to reach a compromise of economic and ecological benefits.

The sustainable valorization of *L. cubeba* biomass is to extract all components for further transformation into sustainable value-added products without producing any unused wastes, where a simple, green, cost-effective, and scalable separation processes is always the bottleneck for explorations [10]. Pressing is the most common method for lipid extraction because of its convenience in processing a certain amount of oil plants. However, the oil yield is relatively low and the heat generated from prepressing and pressing may increase the possibility of lipid oxidation [11]. Solvent extraction is another traditional method which can increase the oil yield but with concern for the additional steps of solvent removal/recuperation and organic solvent residue [12]. Given this, various emerging techniques have been sprouting based on the green extraction concept and principles [13]. Generally, green extraction advocates using renewable plant resources, innovative technologies, and eco-friendly solvents to reduce unit operations, waste, and energy consumption, resulting in a non-denatured and biodegradable extract without contaminants. Ultrasound is a well-known green extraction technique in the process intensification, which could largely reduce the treatment time, where the implosion of ultrasonic cavitation bubble leads to micro-jetting on the vegetal surface and macro-turbulences in liquid media, resulting in various mechanisms such as fragmentation, erosion, capillarity, detexturation, and sonoporation corresponding to extraction yield increase [14]. Moreover, although supercritical CO_2_ extraction could also be considered a green extraction with a higher extraction rate for *L. cubeba* kernel oils, its industrialization may be not suitable due to the payment imbalance concerning specific operating conditions and equipment required [15]. The effect of alternative eco-friendly solvents on the green extraction of *L. cubeba* kernel oils as new oil sources had been investigated before [7]. Nonetheless, for the residues after *L. cubeba* essential oil production, either they are disposed in time or not handled, queries such as the necessity of peeling off the skin from these dried fruits and the practical feasibility of green processing with appropriate extraction and refining techniques should be emphasized toward a sustainable valorization for future *L. cubeba* industry.

**Figure 1:**
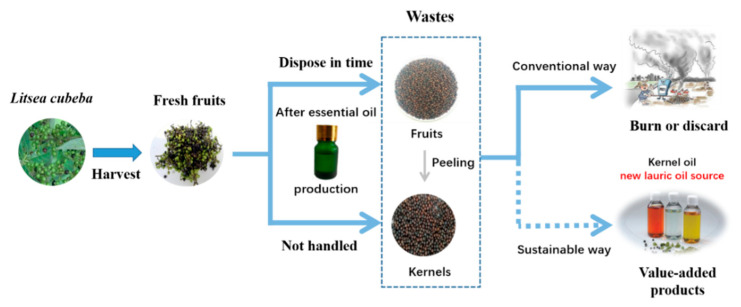
Sustainable valorization pathway proposed for *Litsea cubeba*.

Since there are a few studies dealing with investigation of *L. cubeba* residues (e.g., kernels, peels and fruits), a sustainable valorization pathway was proposed in this work (Figure 1), where the effect of alternative extraction and refining methods on their compositions and properties was originally explored. For *L. cubeba* kernel oils, ultrasound-assisted extraction (UAE) was selected as an alternative green method to conventional solvent extraction (CSE) and cold pressing in terms of acylglycerol and fatty acid composition, physiochemical properties, and total phenolic content (TPC) as well. Furthermore, the resulting *L. cubeba* kernel and fruit oils from cold pressing were further decolorized using activated clay and its combination with alkali deacidification so as to investigate their potential applicability and the effect of refining methods. Last but not the least, no matter which extraction method was used, the characterization of *L. cubeba* kernel oil as the new lauric oil source will provide valuable information for future *L. cubeba* industry to develop a sustainable valorization route toward a zero-waste biorefinery.

## 2. Materials and Methods

### 2.1. Matertials

The sampling area belongs to Zhuang and Yao Autonomous Country in Lianshan (24°57′ N, 112°08′ E) in the North Guangdong, China, which is a sub-tropical humid monsoon climate. The land is fertile, where red loam is the most widely distributed soil type. To guarantee the uniformity of raw materials, residues after *L. cubeba* essential oil production including fruits and kernels collected during the harvest days (July-August) after blossom (March-April) in 2020 were sundried by Qingyuan Yaokang Biotechnology. *L. cubeba* fruits could also be moistened and peeled in order to obtain kernels and peels, which were both oven-dried at 40 °C for 24 h. Dry *L. cubeba* fruits and kernels were pulverized and sieved through a No.60 stainless mesh screen (0.25 mm). The resulting powders were used for moisture and oil content measurement.

Folin-Ciocalteu reagent was purchased from Shanghai Maclean Biotech, Shanghai, China while gallic acid was purchased from Sigma, Louis, MO, USA. Ethanol, isopropanol, n-hexane, glacial acetic acid, chloroform, and potassium hydroxide were from Guangzhou Guanghua Technology, Guangzhou, China. All solvents and chemicals used were of analytical grade. Moreover, virgin coconut oil from local supermarket was used for the following lipid analysis as the reference food grade oil. The main component of food-grade activated clay purchased from Gongyi Haixing Water Supply Material, Zhengzhou, China was bentonite.

### 2.2. Moisture and Oil Content Measurement

The initial moisture and oil content of *L. cubeba* fruits and kernels were measured by SN-720 rapid moisture analyzer (Sanli Chemicals, Shenzhen, China) and Soxhlet extraction as previously reported [7]. Five grams of *L. cubeba* powders were extracted under reflux with 100 mL of n-hexane for 8 h (6–8 cycles/h) in a Soxhlet apparatus (50 mL) fitted with a condenser on a distillation flask (150 mL). The flask was weighed to calculate the oil content after solvent evaporation. The operation above was repeated until the weight variation between two consecutive measurements was less than 10% (*w*/*w*). The oil content was calculated as Equation (1) described,

Oil content (%) = [(Weight of oil obtained after Soxhlet extraction)/(Weight of dry sample for extraction)] × 100(1)

### 2.3. Oil Extractions

**Figure 2:**
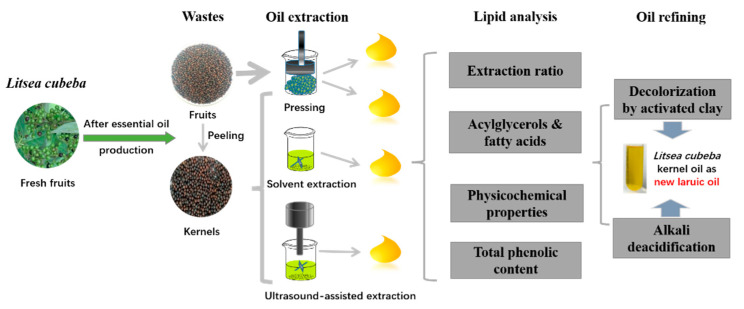
Schematic experimental design.

As depicted in Figure 2, the extraction of lipids from *L. cubeba* residues were conducted, where fruits and kernels were directly pressed on one hand, kernel powders were extracted using CSE and UAE in the meanwhile.

#### 2.3.1. Conventional Extractions

Generally, cold pressing and solvent extraction are two classic conventional lipid extraction methods. On the one hand, two kilograms of *L. cubeba* fruit and kernel were pressed by a CZR091 domestic screw-press (Wanbiao, Guangzhou, China). On the other hand, five grams of *L. cubeba* kernel powders were magnetically stirred with n-hexane in the beaker for a CSE at room temperature. Solid–liquid ratio and extraction time were the same in both CSE and UAE. After extraction, the supernatant was withdrawn after 10 min centrifugation at 3500 rpm. The beaker was weighed to calculate the oil content after solvent evaporation. The operation above was repeated until the weight variation between two consecutive measurements was less than 10% (*w*/*w*). The extraction ratio of both cold pressing and solvent extraction was calculated as Equation (2) described.

Extraction rate (%) = [(Weight of oil obtained after extraction)/(Weight of dry sample before extraction)] × 100(2)

#### 2.3.2. Ultrasound-Assisted Extraction (UAE)

Five grams of *L. cubeba* kernel powders were extracted with n-hexane in ice bath using the JY98-IIIDM ultrasonic cell disruptor (Scientz, Ningbo, China). The single factor experiment was implemented for ultrasonic power (0, 60, 240, 420, 600, and 840 W), solid-liquid ratio (1:5, 1:10, 1:15, 1:20, 1:25), and extraction time (1, 3, 12, 21, and 30 min) in order to obtain the optimal condition for UAE. After ultrasonication, the supernatant was separated for solvent evaporation after centrifugation at 3500 rpm for 10 min, resulting in the final oil for the weight measurement. The weight variation between two consecutive measurements should be less than 10% (*w*/*w*). The extraction rate of oil was obtained as Equation (2) described.

### 2.4. Lipid Analysis

#### 2.4.1. Determination of Physicochemical Properties

DM40 density meter (Mettler Toledo, Switzerland) was used to determine density directly and WYA-2WAJ Abbe refractometer (INESA Instrument, Shanghai, China) was for refractive index. Acidity, peroxide, and saponification value were determined according to AOCS official method Cd 3d-63, Cd 8-53, Cd 3-25, respectively [16,17,18].

Melting and crystallization properties were measured by a differential scanning calorimeter (DSC1, Mettler Toledo, Switzerland) using our developed method [19]. The temperature for the DSC measurements was programmed to heat from initial 25 °C to 80 °C at 40 °C/min first, held for 10 min, and then reduced to −50 °C at 5 °C/min, which was kept for 10 min to obtain the cooling curve. Subsequently, the samples were heated to 80 °C again at 5 °C/min for obtaining the melting curve. According to the cooling and melting curves, the melting point and crystallization onset temperature of samples could be easily obtained.

#### 2.4.2. Acylglycerol and Fatty Acid Composition Analysis

The composition of acylglycerols and fatty acids in *L. cubeba* lipids extracted was analyzed by a 7820A gas chromatography-flame ionization detector (GC-FID) from Agilent Technologies, USA. For acylglycerol composition analysis, a capillary DB-1ht column (15 m × 0.25 mm × 0.1 µm) was used with nitrogen as the carrier gas under a constant flow pressure of 20 psi. All samples dissolved in n-hexane at 10.0 mg/mL were passed through 0.45 µm of filter membranes, of which 0.4 µL was injected at a flow rate of 4.41 mL/min with a split ratio of 40:1. The temperature of both injector and detector was set at 380 °C. The oven temperature was programmed as follows: the initial temperature set at 50 °C was kept for 1 min and heated to 100 °C at 50 °C/min after, then increased to 220 °C at 80 °C/min, and at 15 °C/min from 220 °C to 290 °C. This temperature maintained for 2 min and for 3 min separately increased to 330 °C and to 380 °C at 50 °C/min after. The composition of acylglycerols was expressed as the relative percentages of total acylglycerols.

For fatty acid composition analysis, a DB-wax capillary column (10.0 m × 0.1 mm × 0.2 μm) was used with nitrogen as the carrier gas under a constant flow pressure of 55.7 psi. Fatty acids in 20 mg of oils were transformed to fatty acid methyl esters (FAME) first and samples of 0.5 μL were injected at a flow rate of 15.70 mL/min with a split ratio of 20:1. The temperature of both injector and detector was 240 °C. The oven temperature was programmed to increase from initial 100 to 220 °C at 5 °C/min, held at 220 °C for 2 min, then heated up to 240 °C at 40 °C/min and held for 2 min. Data were collected using Agilent EZChrom Elite software, which could identify FAMEs in comparison with 37 FAME standards (Supelco, Bellefonte, PA, USA). Similar to acylglycerols, the quantification of fatty acid composition was expressed as the relative percentage of total fatty acids.

#### 2.4.3. Total Phenolic Content

Oil samples of 0.5 g were mixed with n-hexane (1.5 mL) and methanolic aqueous solutions (80%) for 5 min in centrifugal tubes, where the supernatant was extracted repeatedly after 10 min centrifugation at 5000 rpm, and the subnatant was combined for the TPC determination of *L. cubeba* fruit and kernel oils at the absorbance of 765 nm according to the Folin-Ciocalteu colorimetric assay. The TPC was expressed as mg of gallic acid equivalent per gram of dried weight (mg GAE/g dw) after measurements.

### 2.5. Oil Refining

#### 2.5.1. Decolorization by Activated Clay

Oil samples of 10 g were magnetically stirred with a certain proportion (5%, 10%, 15%, and 20%) of activated clay at 105 °C for 30 min. The mixture was centrifuged for 10 min at 3500 rpm, resulting the supernatant (i.e., decolorized oils). The yield of decolorized oils could be calculated by the Equation (3),

Yield (%) = [(Weight of oil obtained after decolorization)/(Weight of oil samples before decolorization)] × 100(3)

The color of decolorized oils was measured by a WSL-2 Colorimeter (INESA, Shanghai, China) according to the AOCS official method Cc 13a-43 [20]. The value of a*, b*, and c* of oil samples was obtained to indicate redness, yellowness, and blueness, respectively.

#### 2.5.2. Alkali Deacidification

The alkali deacidification of *L. cubeba* kernel oils was also attempted with 10 g of oil samples reacted with the dropwise addition of alkali solutions at 70 °C for 4 min. The mixture (2.5 g) of n-hexane and 95% ethanol (1:1, *w*/*w*) was then added as the extractant and stirred for 2 min, the resulting reactant was centrifuged for 5 min at 5000 rpm, where the supernatant was removed and evaporated for weight measurement. The operation above was repeated until the weight variation between two consecutive measurements was less than 10% (*w*/*w*). The alkali amount dissolved in 1 mL of deionized water was calculated by Equation (4) and the yield of the deacidified oil was calculated according to Equation (5).

m_KOH_ = Acid value × 1.05 × m_oil_ (4)

Yield (%) = [(Weight of oil obtained after deacidification)/(Weight of oil samples before deacidification)] × 100(5)

### 2.6. Statistical Analysis

All experiments were performed in triplicate, which data were expressed as mean ± standard deviation. The statistical analysis of variance (ANOVA) was determined using the Tukey’s test in the IBM SPSS Statistics 22 software. Significant differences (*p* < 0.05) at 95% level between samples tested were labeled by different superscript letters.

## 3. Results and Discussions

### 3.1. Optimization of UAE Parameters

**Figure 3:**
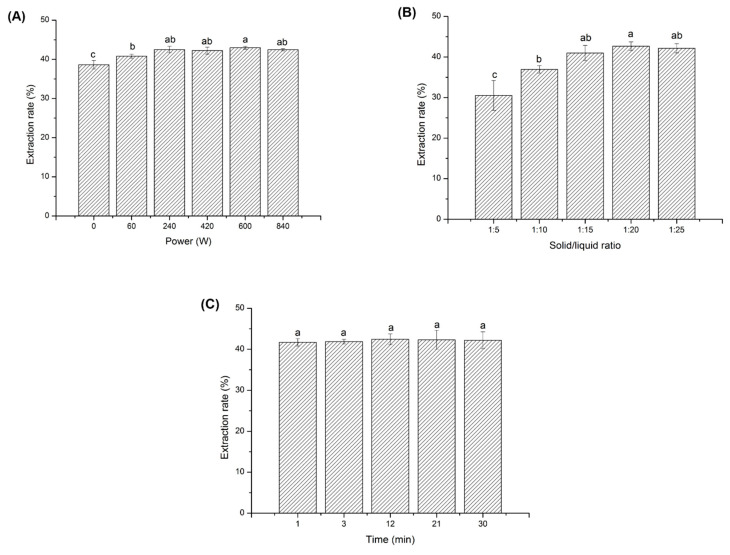
Single factor effect on the extraction rate of ultrasound-assisted extraction of *Litsea cubeba* kernel oils: (**A**) ultrasonic power, (**B**) solid–liquid ratio, and (**C**) extraction time. Columns marked by the same letter are not significantly at *p* < 0.05.

The initial oil and moisture content demonstrated that *L. cubeba* kernels contained most oils (43.80 ± 0.92%) but less water (6.99 ± 0.15%) as compared to that in *L. cubeba* fruits (39.33 ± 2.15% for lipid and 8.28 ± 0.2% for moisture). The higher moisture content also indicates that *L. cubeba* fruits are not conducive to the long-term storage due to the fact that adequate water content and time in any raw materials accelerate enzymatic reaction rate and microbial spoilage [21]. The effect of single factor on the extraction rate of UAE was illustrated in Figure 3. Different ultrasonic power was studied first while other variables were set as 10 min and 1:25. Ultrasound could effectively improve the extraction rate of *L. cubeba* kernel oil compared to normal extraction without ultrasonic assistance (38.63 ± 1.09%). This could be explained by the fact that acoustic cavitation can accelerate the cell wall disruption in order to improve the mass diffusivity and solvation properties of the solvent [15]. However, the growth of extraction rate was not significant as the ultrasonic power increased higher than 240 W (Figure 3A), which was chosen throughout the following experiments. As shown in Figure 3B, the extraction rate of *L. cubeba* kernel oil reached the maximum (42.66 ± 1.04%) due to the increasing mass transfer efficiency when the solid: liquid ratio increased from 1:5 to 1:20 [22]. Most oils were extracted at this moment so that the extraction rate after this peak approached to a flat. Hence, the solid: liquid ratio of 1:20 was used for the following extractions from a cost-effective point of view. At last, no significant difference was found for the time effect on the extraction rate of *L. cubeba* kernel oil when UAE was implemented using ultrasonic power of 240 W and solid: liquid ratio of 1:20 (Figure 3C). Surprisingly, the extraction of most oils could be completed in 1 min, which might indicate that the structure of *L. cubeba* kernels after pretreatment was soft and suitable for ultrasonic treatment, and its mechanical resistance level to ultrasonication was very low [23]. Given the above, the optimal parameters for UAE were determined as 240 W for ultrasonic power, 1:20 for solid–liquid ratio and 1 min for extraction time with the consideration of extraction efficiency, solvent consumption, cost, and energy saving, which were used in subsequent extractions of *L. cubeba* kernel oils.

### 3.2. The Effect of Extraction Methods on the Extraction Rate of L. cubeba Kernel and Fruit Oils

**Figure 4:**
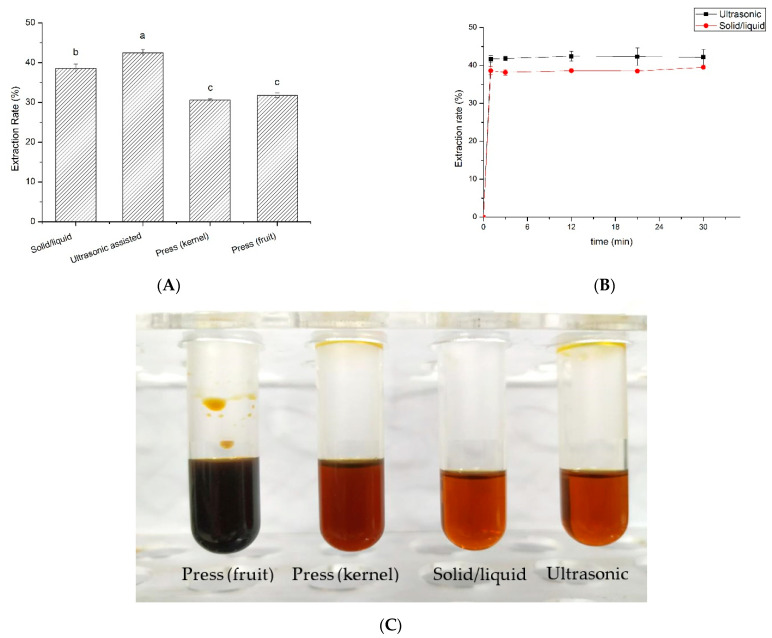
(**A**) The effect of extraction methods on the extraction rate of *Litsea cubeba* oils, columns marked by the same letter are not significant at *p* < 0.05. (**B**) Kinetics comparison of conventional solid–liquid extraction and ultrasound-assisted extraction as the function of extraction time, (**C**) the appearance of *Litsea cubeba* oils extracted from different methods.

The effect of alternative extraction methods on the lipid extraction from *L. cubeba* kernels and fruits is shown in Figure 4. First, no significant difference was found in the extraction rate of pressing between *L. cubeba* fruit and kernel oils. Compared to pressing, CSE and UAE obtained higher extraction rate, where UAE under optimal conditions achieved the highest (42.48 ± 0.83%). Moreover, the light-colored appearance of *L. cubeba* kernel oils seems more desirable than that of fruit oils. Amazingly, as the extraction time increased, the extraction rate of CSE showed a similar growing trend to that of UAE though they had a significant difference in between. Moreover, both CSE and UAE reached to a certain extraction rate in only 1 min and remained nearly stable afterwards, indicating that ultrasound could indeed increase the extraction rate of CSE, which is in consistency with most UAE studies. Nevertheless, it is worthy to note that the structure of *L. cubeba* kernels seems much softer than other soft oilseeds [24,25], which was favorable to lipid extraction using hexane no matter which extraction method used. Hence, UAE might be a good alternative to conventional extraction method for *L. cubeba* kernel oils.

### 3.3. Analysis of LC Kernel and Fruit Oils Extracted by Different Methods

#### 3.3.1. Acylglycerol and Fatty Acid Composition

As can be seen in Table 1, similar acylglycerol and fatty acid compositions were found in *L. cubeba* kernel oils extracted by different extraction methods except for *L. cubeba* fruit oils. Compared to cold pressed *L. cubeba* kernel oils, CSE and UAE could extract more diglycerides (DAG) and monoglycerides (MAG) in *L. cubeba* kernel oils but with nearly the same level of triglycerides (TAG) and free fatty acids (FFA). *L. cubeba* kernel oils contained more than 86% of TAG and around 2.7% of FFA while its fruit oils contained much lower TAG (69.44 ± 1.50%) but nine times higher FFA (23.32 ± 1.55%). Hence, how to reduce the FFA content is the key for further deep processing of *L. cubeba* oils by referring to edible oils with a low free acidity ranging from 0.62 to 1.21% [26]. Moreover, medium chain fatty acids have fast absorption and metabolism rates in vivo, medium chain triglycerides (MCT) were reported to have the potential to prevent lipid accumulation [27]. It is noticeable that medium chain triglycerides (MCT) such as tri-saturated C_10_C_12_C_12_ and C_12_C_12_C_12_ in *L. cubeba* kernel oils had a total proportion of more than 65%, which is higher than that in common lauric oils like palm kernel oil and coconut oil [28]. Compared to virgin coconut oil, *L. cubeba* kernel oil had not only a comparable lauric acid content, but also a higher unsaturation degree, particularly for essential fatty acid such as linoleic acid. Although the fatty acid composition of laurel fruits was found to be influenced by growth conditions and collection site, the lauric acid content of *L. cubeba* kernel oil is more than double of that in *Laurus nobilis* fruit oils [29]. As compared to other characteristic plant oils, avocado oil obtained by ultrasound-assisted cold extraction showed a balanced composition in saturated, monounsaturated, and polyunsaturated fatty acids, where γ-linolenic, α-linolenic, conjugated α-linolenic, palmitic, and even palmitoleic acids were identified with a considerable content [30]. Although *L. cubeba* kernel oil does not have essential fatty acid content as high as avocado and tomato seed oil [31], its medium-chain monounsaturated fatty acids are much more abundant, some uncommon fatty acids such as lauroleic acid, caproleic acid, and myristoleic acid deserve to be further explored for their undefined functions. Based on the above, *L. cubeba* kernel oil could be used as a new lauric oil source such as existing edible oils, but it is highly recommended for use in soaps, massage oils, creams, and shampoo collocating with its homologous essential oil in a well formulated recipe. Moreover, several edible vegetable oils (i.e., olive oil, peanut oil, and sunflower oil) with higher unsaturated degrees had reported their suitability for biodiesel production with respect to the relevant standards [26,32,33,34]. Considering the competition to food supply and the unique medium-chain saturated fatty acid composition in *L. cubeba* kernel oil, using *L. cubeba* kernel oil as the feedstock for biolubricant base oil production had also been successfully achieved with good oxidative stability and low-temperature performance [8], which may pave the way for future biofuel applications.

#### 3.3.2. Physicochemical Properties

As can be seen in Table 2, there was no significant differences between CSE and UAE in physicochemical properties of *L. cubeba* kernel oils, whereas pressed kernel oils showed a significant variation in several indicators. Pressed oils could maintain more micro-ingredients resulting in a significantly higher value of density, refractive index, acid value and color, and lower peroxide value and crystallization onset temperature. Nonetheless, no significant difference was found in kernel oils concerning melting point, saponification value, and crystallization enthalpy among three extraction methods.

As compared to virgin coconut oil, pressing fruits could directly extract both free fatty acids and some antioxidant components from peels, resulting in a lowest peroxide value, which is in accordance with its higher total phenolic content [35]. Moreover, the lower saponification value of *L. cubeba* fruit oil could be explained by its impurity and fatty acid composition (i.e., more palmitic acid and less lauric acid). Cold pressing seems to preserve more phenolic compounds in *L. cubeba* kernel and fruit oils, which is beneficial to oil stability [36]. However, their higher acid values and peroxide values, as well as the variation in melting and crystallization properties, should be taken into consideration for further refining to widen its industrial application.

Moreover, the significant difference in fatty acid composition between *L. cubeba* fruit and kernel oils was found to be attributed to the abundant free fatty acids in *L. cubeba* peels. According to the Figure S1, the main difference between gas chromatogram of *L. cubeba* fruit and kernel oils was the peaks at around 4 min representing free fatty acids, which was in accordance with that of *L. cubeba* peels at the same retention time. Around 97% of free fatty acids were quantified in *L. cubeba* peels, mainly including palmitic acid (29%), oleic acid (29%), and linoleic acid (39%), which could be extracted along with the oil expression. Regardless of food or nonfood application for *L. cubeba* oil, *L. cubeba* kernel without peels seems more suitable for direct oil pressing and further processing because of the relatively acceptable physiochemical properties of its oils. However, *L. cubeba* peel might be a potential natural feedstock for the extraction of its fatty acids so as to improve its utilization rate and added value.

### 3.4. Refining of Pressed L. cubeba Fruit and Kernel Oils

**Figure 5:**
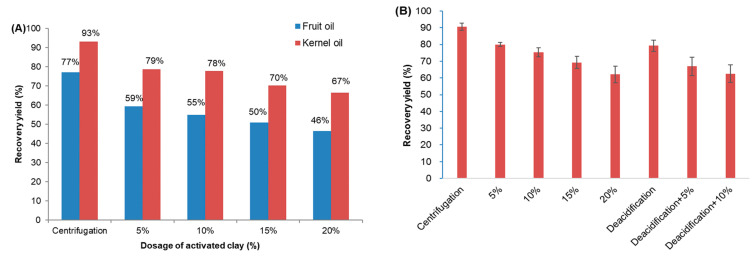
The recovery yield of *Litsea cubeba* fruit and kernel oils after decoloration using (**A**) activated clay only and (**B**) alkali deacidification combined with using activated clay.

Considering the results above, pressed *L. cubeba* fruit and kernel oils were selected for refining process toward the consumer demand of naturalness. Color is one of the evaluation indicators for the quality of oils used in cosmetics, fine chemicals, or even food industries, where light-colored oils are more desirable. At present, activated clay has become the most widely used decolorizing agent for edible oils due to its large surface area, strong adsorption, and high chemical inertness. Thus, decolorization using activated clay was first attempted as Figure S2 illustrated, it is interesting to note that no decoloring effect was observed on *L. cubeba* fruit oils whereas remarkable effect was found for the decoloration of *L. cubeba* kernel oils, which showed satisfying pale yellow when the dosage of activated clay was 20% wt. Notwithstanding, the recovery yield of *L. cubeba* oils after such decoloration dramatically reduced as the dosage of activated clay increased (Figure 5A), which may be due to the fact that activated clays could absorb other components other than pigments. Furthermore, the acid value of *L. cubeba* fruit oil rose up with the increasing dosage of activated clay while no significant change was found for *L. cubeba* kernel oils (Table 3). From this, *L. cubeba* fruit oil was not suitable for further processing due to its unsatisfactory decoloring effect and acid value. In contrast, *L. cubeba* kernel oils may have the potential for further applications.

**Figure 6:**
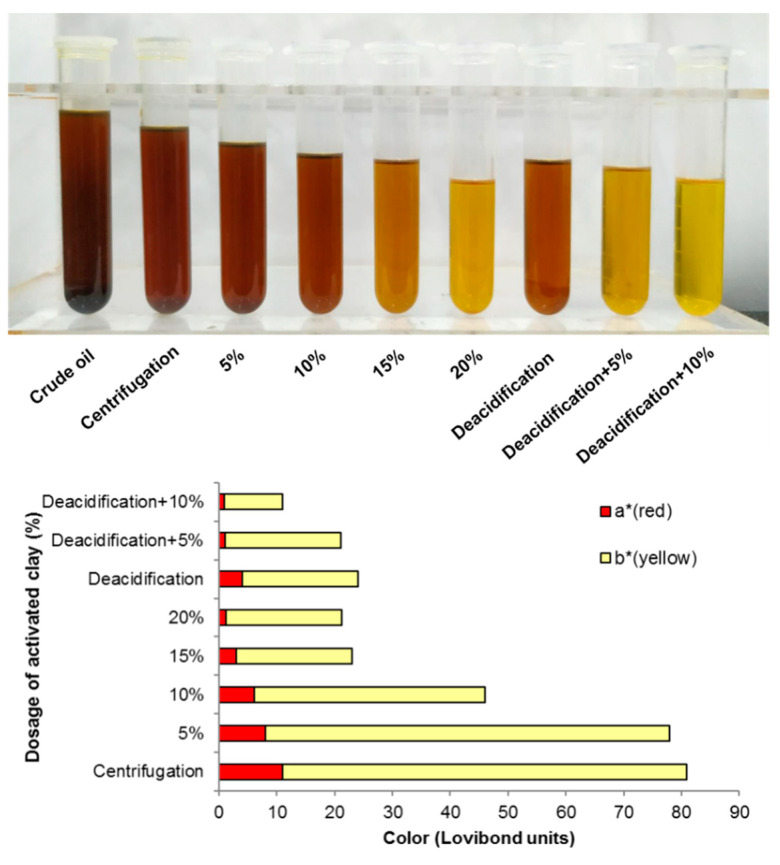
The decoloring effect of different refining methods on *Litsea cubeba* kernel oils.

Generally, crude plant oils will conduct deacidification before decolorization, which also has a certain decoloring effect. As Figure 6 presented, the combination of alkali deacidification with activated clay showed a better decoloring effect than decolorization using activated clay solely, which could largely reduce the dosage of activated clay. Alkali deacidification with 10% of activated clay performed the best decoloring effect, in which recovery yield is equivalent to that of decolorization using 20% of activated clay only (Figure 5B). The effect of different refining methods on the basic physicochemical properties of *L. cubeba* kernel oils could be seen in Table 3. Deacidification could reduce the acid value and peroxide value to the acceptable level for further application of *L. cubeba* kernel oils as exotic oils [37]. The addition of activated clay could significantly increase the acid value and peroxide value of deacidified *L. cubeba* kernel oils but no dose-dependent manner was hereby found. Activated clay could absorb more pigments accompanying with bioactive phenolic compounds as dosage increased, resulting in a higher peroxide value. Based on the similar decoloring effect and recovery yield, the TPC of deacidified oils using 10% of activated clay was lower than that of decolored oils using 20% of activated clay. In brief, both alkali deacidification and decoloration using activated clay could achieve acceptable decoloring effect, where the former could save dosage of activated clay by 50–75% though they both caused a significant loss of TPC. After all, deacidification with 10% of activated clay seems to be the good alternative refining method for further *L. cubeba* kernel oil processing.

## 4. Conclusions

The effect of alternative green extraction and refining methods on *L. cubeba* residues toward the sustainable valorization was originally investigated in this study. Compared to most plant oils, *L. cubeba* kernel oil with a higher content of medium fatty acid chains was demonstrated as a new promising lauric oil with a higher saturation level (≈74%). The extraction rate of UAE was better than conventional pressing and solvent extraction, which was proved to be as an alternative method due to the fact that no significant difference was noticed between the different extraction techniques regarding some physiochemical properties such as acylglycerol composition, density, refractive index, and melting point. Moreover, *L. cubeba* peels were demonstrated as a promising source for natural fatty acids such as palmitic, oleic and linoleic acids, resulting in a higher acid value in *L. cubeba* fruit oils than that in *L. cubeba* kernel oils. *L. cubeba* fruit oils with deeper color and higher viscosity were difficult to be refined by decolorization though they had a high TPC. The decoloration and deacidification of *L. cubeba* kernel oils could be achieved to some extent by using either activated clay solely or its combination with alkali deacidification. However, the recovery yield of *L. cubeba* kernel oils with optimum refining effect was relatively low accompanying with the loss of bioactive compounds such as phenolics. From the practical point of view, *L. cubeba* fruit oil is not suitable for further refining and applications whereas mild refining process is recommended for *L. cubeba* kernel oils to maintain bioactive minor compounds at a desirable level. The result of this study is of great practical significance, which could provide a valuable reference for future sustainable valorization of similar woody plant resources for the cleaner production of various plant-based products without any wastes.

## Figures and Tables

**Table 1:** The effect of different extraction methods on the composition of acylglycerols and fatty acids in *Litsea cubeba* oils.

*Litsea cubeba*	Kernel			Fruit	
	Conventional Solvent Extraction	Ultrasound-Assisted Extraction	Cold Pressing	Cold Pressing	Virgin Coconut Oil
Acylglycerols (%)					
TAG	86.55 ± 0.09 ^a^	86.08 ± 0.06 ^a^	89.10 ± 0.10 ^a^	69.44 ± 1.50 ^b^	94.52 ± 0.28
C_10_C_12_C_12_	31.66 ± 0.05 ^a^	31.45 ± 0.03 ^a^	29.69 ± 0.05 ^b^	21.60 ± 0.24 ^c^	17.07 ± 0.11
C_12_C_12_C_12_	31.50 ± 0.05 ^b^	31.31 ± 0.17 ^b^	33.14 ± 0.01 ^a^	24.17 ± 0.58 ^c^	19.91 ± 0.01
DAG	10.00 ± 0.40 ^a^	10.38 ± 0.04 ^a^	7.77 ± 0.03 ^b^	6.41 ± 0.01 ^c^	13.92 ± 0.07
MAG	0.73 ± 0.19 ^ab^	0.83 ± 0.01 ^a^	0.32 ± 0.11 ^b^	0.83 ± 0.04 ^a^	-
FFA	2.72 ± 0.31 ^b^	2.71 ± 0.02 ^b^	2.81 ± 0.17 ^b^	23.32 ± 1.55 ^a^	0.08 ± 0.03
Fatty acids (%)					
Saturated					
Octanoic acid (C8:0)	-	-	-	-	5.07 ± 0.02
Capric acid (C10:0)	15.28 ± 2.68 ^a^	13.37 ± 1.40 ^a^	11.26 ± 0.82 ^a^	7.03 ± 0.51 ^b^	5.54 ± 0.11
Lauric acid (C12:0)	55.34 ± 3.15 ^a^	57.16 ± 0.20 ^a^	57.39 ± 0.84 ^a^	31.92 ± 1.20 ^b^	50.60 ± 0.19
Myristic acid (C14:0)	2.97 ± 0.77 ^a^	3.15 ± 0.59 ^a^	2.47 ± 0.42 ^a^	1.71 ± 0.31 ^a^	19.88 ± 0.43
Palmitic acid (C16:0)	2.68 ± 0.27 ^b^	2.54 ± 0.22 ^b^	3.61 ± 0.52 ^b^	14.55 ± 1.36 ^a^	9.18 ± 0.30
Stearic acid (C18:0)	-	-	-	-	4.13 ± 0.74
Mono-unsaturated					
Caproleic acid (C10:1)	1.09 ± 0.37 ^ab^	0.49 ± 0.05 ^b^	1.40 ± 0.52 ^a^	0.72 ± 0.21 ^ab^	-
Lauroleic acid (C12:1)	6.62 ± 0.29 ^a^	7.05 ± 0.62 ^a^	5.89 ± 1.07 ^a^	3.69 ± 0.31 ^b^	-
Myristoleic acid (C14:1)	1.21 ± 0.06 ^a^	1.36 ± 0.31 ^a^	1.30 ± 0.38 ^a^	1.15 ± 0.59 ^a^	-
Palmitoleic acid (C16:1)	-	-	-	1.77 ± 0.30	-
Oleic acid (C18:1)	9.59 ± 1.07 ^b^	9.57 ± 0.48 ^b^	10.90 ± 0.69 ^b^	19.07 ± 1.31 ^a^	5.61 ± 0.05
Poly-unsaturated					
Linoleic acid (C18:2)	5.23 ± 0.45 ^b^	5.31 ± 0.62 ^b^	5.78 ± 0.20 ^b^	18.40 ± 0.52 ^a^	-
∑SFAs	76.27 ± 1.29 ^a^	76.21 ± 0.94 ^a^	74.73 ± 1.05 ^a^	55.21 ± 1.81 ^b^	94.39 ± 0.05
∑UFAs	23.74 ± 1.46 ^b^	23.79 ± 1.44 ^b^	25.27 ± 1.06 ^b^	44.80 ± 2.17 ^a^	5.61 ± 0.05
∑EFAs	5.23 ± 0.45 ^b^	5.31 ± 0.62 ^b^	5.78 ± 0.20 ^b^	18.40 ± 0.52 ^a^	-

SFAs: saturated fatty acids, UFAs: unsaturated fatty acids, EFAs: essential fatty acids. Values followed by the same letter in the same row are not significant at *p* < 0.05, which are presented as mean ± standard deviation of triplicate.

**Table 2:** The effect of different extraction methods on the physicochemical properties of *Litsea cubeba* oils.

*Litsea cubeba*	Kernel			Fruit	
	Conventional Solvent Extraction	Ultrasound-Assisted Extraction	Cold Pressing	Cold Pressing	Virgin Coconut Oil
Density (g/cm^3^)	0.92 ± 0.00 ^b^	0.92 ± 0.00 ^b^	0.94 ± 0.01 ^a^	0.95 ± 0.00 ^a^	0.91 ± 0.01
Refractive index (20 °C)	1.46 ± 0.00 ^c^	1.46 ± 0.00 ^c^	1.47 ± 0.00 ^b^	1.48 ± 0.00 ^a^	1.43 ± 0.00
Acid value (mg KOH/g)	9.93 ± 0.16 ^c^	9.62 ± 0.09 ^c^	12.13 ± 0.35 ^b^	58.76 ± 0.39 ^a^	0.43 ± 0.02
Peroxide value (meq O_2_/kg)	1.31 ± 0.02 ^a^	1.32 ± 0.25 ^a^	0.54 ± 0.01 ^b^	0.18 ± 0.02 ^c^	0.11 ± 0.01
Saponification value (mg KOH/g)	297.55 ± 5.81 ^a^	295.90 ± 4.67 ^a^	287.71 ± 6.13 ^a^	268.42 ± 5.35 ^b^	299.64 ± 4.74
Melting point (°C)	28.60 ± 0.27 ^a^	28.29 ± 0.18 ^a^	28.49 ± 0.11 ^a^	23.40 ± 0.53 ^b^	25.17 ± 0.64
Crystallization onset temperature (°C)	−1.73 ± 0.24 ^ab^	−1.69 ± 0.14 ^a^	−2.40 ± 0.06 ^b^	−1.29 ± 0.33 ^a^	3.81 ± 0.40
Total △Hc (J/g)	54.14 ± 4.57 ^a^	54.56 ± 2.73 ^a^	53.41 ± 4.92 ^a^	25.50 ± 0.95 ^b^	39.10 ± 1.58
Color (Lovibond units)					
a* (red/green value)	6	6.1	10.9	6	
b* (yellow/blue value)	40	40	70	60	
L* (brightness value)	-	-	-	13	
Total phenolic content (mg GAE/100 g)	43.22 ± 1.94 ^c^	43.37 ± 1.77 ^c^	72.79 ± 1.04 ^b^	176.14 ± 4.81 ^a^	15.91 ± 3.85 ^d^

Values followed by the same letter in the same row are not significantly at *p* < 0.05, which are presented as mean ± standard deviation of triplicate.

**Table 3:** The effect of different refining methods on the basic physicochemical properties of *Litsea cubeba* kernel and fruit oils.

The Effect of Decoloration Using Activated Clay on the Acid Value					
***Litsea cubeba***	Centrifugation	5%	10%	15%	20%
Fruit oil	58.76 ± 0.39 ^c^	58.02 ± 0.05 ^c^	58.97 ± 0.28 ^bc^	60.09 ± 0.41 ^ab^	60.60 ± 0.41 ^a^
Kernel oil	11.68 ± 0.15 ^a^	11.60 ± 0.11 ^a^	11.55 ± 0.03 ^ab^	11.20 ± 0.07 ^c^	11.35 ± 0.10 ^bc^
Basic physicochemical properties of *Litsea cubeba* kernel oils					
Refining methods	Acid value (mg KOH/g)	Peroxide value (meq O_2_/kg)	Total phenolic content(mg GAE/100 g)		
Centrifugation	11.68 ± 0.15 ^a^	0.64 ± 0.18 ^c^	81.29 ± 5.06 ^a^		
5%	11.60 ± 0.11 ^a^	1.27 ± 0.44 ^ab^	49.50 ± 3.97 ^b^		
10%	11.55 ± 0.03 ^ab^	1.82 ± 0.30 ^a^	36.67 ± 2.37 ^c^		
15%	11.20 ± 0.07 ^c^	1.72 ± 0.09 ^a^	22.73 ± 5.61 ^d^		
20%	11.35 ± 0.10 ^bc^	1.51 ± 0.03 ^ab^	17.21 ± 5.03 ^d^		
Deacidification	0.86 ± 0.01 ^e^	0.93 ± 0.15 ^bc^	36.17 ± 6.01 ^c^		
Deacidification + 5%	1.53 ± 0.10 ^d^	1.71 ± 0.05 ^a^	19.00 ± 4.04 ^d^		
Deacidification + 10%	1.57 ± 0.06 ^d^	1.57 ± 0.05 ^a^	11.10 ± 1.26 ^d^		

Values followed by the same letter in the same row are not significantly at *p* < 0.05, which are presented as mean ± standard deviation of triplicate.

## Data Availability

Data are contained within the article.

## Supplementary Materials

The following supporting information can be downloaded at: https://www.mdpi.com/article/10.3390/foods11142047/s1. Figure S1: The gas chromatogram (a) and the compositions of acylglycerols (b) and fatty acids (c) of *Litsea cubeba* (A) peel, (B) fruit, and (C) kernel oils from Soxhlet extraction. Numbers in the concentric circles represent the relative percentages. Figure S2: The decoloring effect of different dosages of activated clay on *Litsea cubeba* oils from (a) fruits and (b) kernels.
