# Supplementary material for: Sustainable Valorization of Litsea cubeba (Lour.) Pers. Residue as the New Lauric Oil Source Using Alternative Green Extraction and Refining Methods
Source: Foods. 2022 Jul 11;11(14):2047. doi: 10.3390/foods11142047 (PMC9324626; doi:10.3390/foods11142047)
Supplement: Supplementary file 1 [file foods-11-02047-s001.zip › foods-1780577-supplementary.pdf]

Supplementary materials

Figure S1. The gas chromatogram (a) and the compositions of acylglycerols (b) and fatty acids (c) of *Litsea cubeba* (A) peel, (B) fruit and (C) kernel oils from Soxhlet extraction. Numbers in the concentric circles represent the relative percentages.

Figure S2. The decoloring effect of different dosages of activated clay on *Litsea cubeba* oils from (a) fruits and (b) kernels.

Figure S1

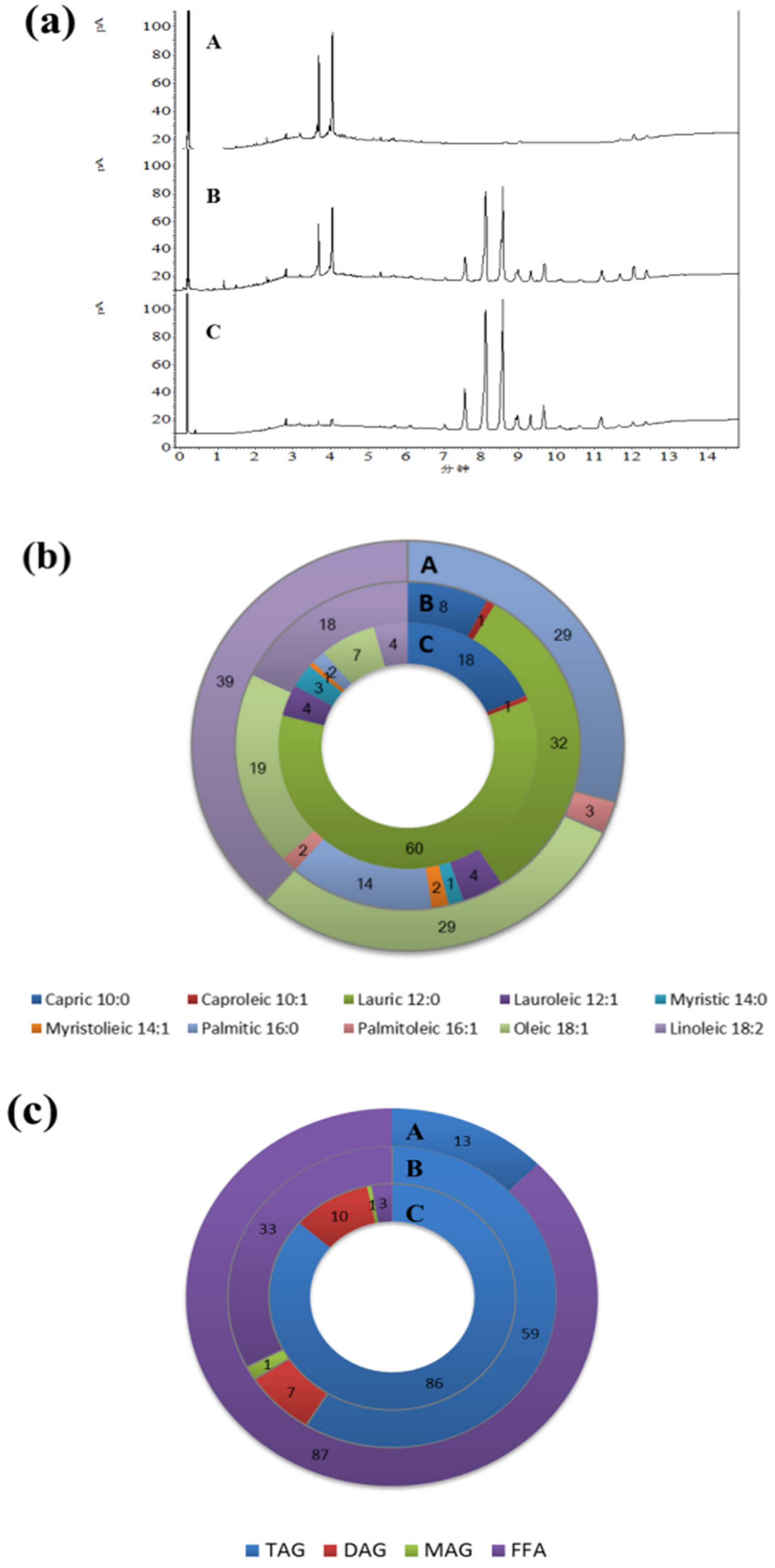

(a)
pA
A
B
C
分钟
(b)
A
B
C
Capric 10:0
Caproleic 10:1
Lauric 12:0
Lauroleic 12:1
Myristic 14:0
Myristolieic 14:1
Palmitic 16:0
Palmitoleic 16:1
Oleic 18:1
Linoleic 18:2
(c)
A
B
C
TAG
DAG
MAG
FFA

Figure S2

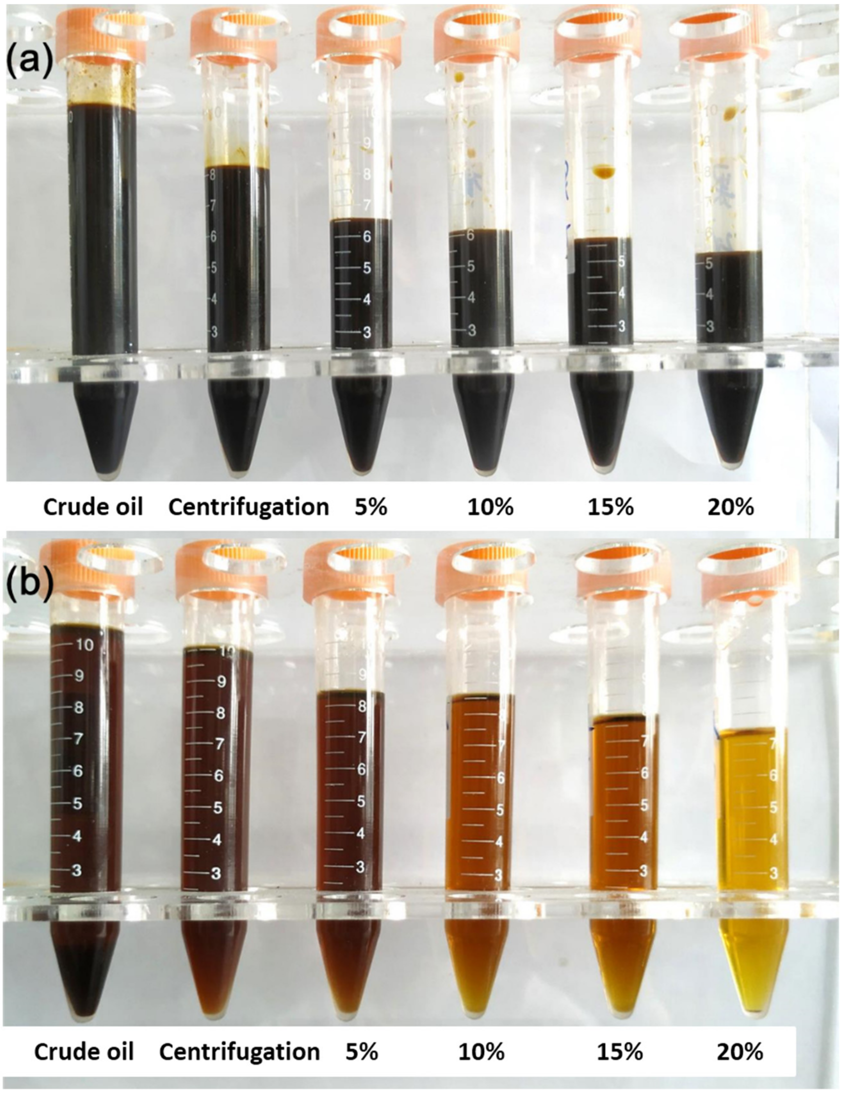
(a)
Crude oil
Centrifugation
5%
10%
15%
20%
(b)
Crude oil
Centrifugation
5%
10%
15%
20%
